# *Staphylococcus sciuri* Strain LCHXa is a Free-Living Lithium-Tolerant Bacterium Isolated from Salar de Atacama, Chile

**DOI:** 10.3390/microorganisms8050668

**Published:** 2020-05-05

**Authors:** Camila Salazar-Ardiles, Tamara Caimanque, Alexandra Galetović, Claudia Vilo, Jorge E. Araya, Nataly Flores, Benito Gómez-Silva

**Affiliations:** 1 Laboratorio de Bioquímica, Departamento Biomédico, and Centre for Biotechnology and Bioengineering, CeBiB, Universidad de Antofagasta, Antofagasta 1270300, Chile; nickolsalazar@gmail.com (C.S.-A.); tamara.caimanque@hotmail.com (T.C.); alexandra.galetovic@uantof.cl (A.G.); claudiavilo@gmail.com (C.V.); nataly.flores@uantof.cl (N.F.); 2 Laboratorio de Parasitología y Biología Molecular, and Centre for Biotechnology and Bioengineering, CeBiB, Universidad de Antofagasta, Antofagasta 1270300, Chile; jorge.araya@uantof.cl

**Keywords:** antibiotic susceptibility, Atacama Desert, Laguna Chaxa, lithium tolerance, Novobiocin resistance, osmoregulation, *Staphylococcus sciuri*, Salar de Atacama

## Abstract

In addition to the industrial and biomedical applications of lithium, information on the tolerance of microorganisms to high Li concentrations in natural biological systems is limited. Strain LCHXa is a novel free-living Gram-positive, non-motile bacterium strain isolated from water samples taken at Laguna Chaxa, a non-industrial water body with the highest soluble Li content (33 mM LiCl) within the Salar de Atacama basin in northern Chile. Enrichment was conducted in Luria-Bertani (LB) medium supplemented with 1 M LiCl. Strain LCHXa was a Novobiocin-resistant and coagulase negative *Staphylococcus*. Phylogenetically, strain LCHXa belongs to the species *Staphylococcus* *sciuri*. Strain LCHXa grew optimally in LB medium at pH 6–8 and 37 °C, and it was able to sustain growth at molar Li concentrations at 2 M LiCl, with a decrease in the specific growth rate of 85%. Osmoregulation in strain LCHXa partially involves glycine betaine and glycerol as compatible solutes.

## 1. Introduction

The Atacama Desert in northern Chile is a coastal hyperarid territory and probably the oldest dryland on our planet [1]. Previously considered a sterile, mineral-rich region with Mars-like soils, microbiological and metagenomic studies in the Atacama have demonstrated an unexpected diversity of microbial life, including lithobionts, colonizing diverse habitats, with many unculturable microorganisms living under continuous desiccation and high solar radiation [2,3,4].

Exploitation of metallic and non-metallic resources in the Atacama has a centuries-long history. Salar de Atacama is a non-fossil evaporitic basin of 14,800 km^2^, located at the eastern border of the Atacama Desert in northern Chile, between the Domeyko and Andes Mountain ranges, at 2300 m above sea level. This basin contains a lithium-rich core of 900 m deep and 1100 km^2^ [5] and represents one of the largest lithium reserves in our planet; together with Salar de Uyuni (Bolivia) and Salar Hombre Muerto (Argentina), they constitute the so-called “Triangle of Lithium” [6].

In addition to some well-known industrial applications of lithium [7], the impact and tolerance of microorganisms to high Li concentrations in natural biological systems is limited; however, the interest on these type microorganisms is increasing [8,9,10,11,12,13,14]. Considering that the natural molar concentration of LiCl represents an environmental chemical stress to microorganisms, several questions can be addressed, and this study was focused on the following two: which are the microorganisms living at natural waters with high Li concentration at Salar de Atacama? And what is the microbial osmoregulatory strategy against high Li concentrations?

During our 2014 sampling campaign at and around Salar de Atacama, we were focused on the isolation of lithium-tolerant microorganisms in non-industrial, natural sites. We were able to isolate several microorganisms after enrichment in a growth medium supplemented with 1 M LiCl. Here, we report the characterization of LCHXa, a free-living *Staphylococcus sciuri* strain LCHXa [14] isolated from Laguna Chaxa, a natural saline water body with the highest soluble Li content (33 mM LiCl) within the Salar de Atacama basin, which was able to sustain growth at molar Li concentrations; also, we provide an insight on key intracellular compatible solutes involved in osmoregulation.

## 2. Materials and Methods

*Sampling site, isolation, and growth of strain LCHXa*. Laguna Chaxa has a surface of 13 km^2^, and it is the major NaCl-dominated natural salty lake, with the highest Li concentration (1393 mg/L) within the Salar de Atacama. Chaxa sustains a variety of wildlife, has a higher primary productivity than other water bodies in the basin, and its chemical and isotopic composition is well characterized [15]. Triplicate samples of water (1 mL) and upper sediment (tablespoon) were taken at the edge of Laguna Chaxa (22°20′ S; 60°35′ W) and immediately inoculated into tubes containing 10 mL of Luria-Bertani (LB) medium or 10 mL of LB medium supplemented with 1 M LiCl (Li-LB). Tubes were taken to the laboratory and incubated at 30 °C until an increase in turbidity was observed (8–15 d). Next, 10 µL aliquots (without or after 1:100 dilution) were seeded onto agar plates, prepared with Li-LB medium, and were incubated at 30 °C until isolated colonies were obtained after approximately 15 d. Isolated colonies were maintained in liquid and solid LB and Li-LB media. Further work was done with a selected identified as strain LCHXa.

*Phenotypic characterization*. Gram staining and cell morphology and color were inspected under a light microscope (100× magnification; MOTIC BA310, Richmond, BC, Canada) [16]. For biochemical tests, isolated colonies were grown and maintained in LB medium (Oxoid) at 30 °C during 18–24 h in a rotary shaker at 120 rpm. Agar plates prepared with the corresponding test media were inoculated, and observations were done after incubation for 18–24 h at 30 °C. Oxoid LB agar medium supplemented with starch, casein, Tween 60, Tween 80 or blood, were used to test exoenzymes (amylase, protease, lipase, DNase) and hemolysis. The catalase test was carried out transferring a colony onto a glass plate followed by the addition of a drop of 30% oxygen peroxide to observe oxygen bubbling. Other tests (oxidase, coagulase, Simmons citrate, LIA, MIO, TSI) were done according to previously reported procedures [17]. The metabolic use of 31 carbon sources was carried out in triplicate using Biolog EcoPlateTM (Hayward, CA, USA), according to the manufacturer’s instructions. Briefly, 200 µL aliquots of an LCHXa culture grown in LB medium for 18 h, were added to each well of the microplate and incubated at 30 °C during 2–5 d. Depending upon the use of a carbon source by the bacterium, a purple color was developed and evaluated at 590 nm.

*Antibiotic susceptibility*. Cells grown for 18–24 h in LB medium or LB containing 1 M NaCl or 1 M LiCl, were diluted with a sterile saline solution according to McFarland scale N° 5 [17] and spread as a lawn onto Mueller-Hinton (MH) agar plates without salt addition or to plates containing 1 M NaCl (MH-Na) or 1 M LiCl (MH-Li). Antibiotic susceptibility was conducted by the disc diffusion test, including 26 antibiotics [18] (Emarin Sda S.A., Ñuñoa, Santiago, Chile) at the following concentrations: Ampicillin/sulbactam (10/10 µg), Lincomycin (2 µg), Clindamycin (2 µg), Penicillin (10 µg), Ampicillin (10 µg), Ceftazidime (30 µg), Fosfomycin (200 µg), Oxacillin (1 µg ug), Amoxicillin/ac. clavulanic (20/10 µg), Novobiocin (5 µg), Cefuroxime (30 µg), Gentamicin (10 µg), Tetracycline (30 µg), Vancomycin (30 µg), Cotrimoxazole (25 µg), Erythromycin (15 µg), Ciprofloxacin (5 µg), Chloramphenicol (30 µg), Neomycin (30 µg), Nitrofurantoin (300 µg), Cefadroxil (30 µg), Streptomycin (300 µg), Levofloxacin (5 µg), Teicoplanin (30 µg), Linezolid (30 µg), and Amikacin (30 µg). Discs were pressed on the agar, left for 10–15 min to dry, and incubated at 35 °C for 18–24 h. Diameters of the inhibition zone were measured and compared with the data table from [18].

*Physiological characterization*. Cells were grown LB medium, and triplicates of 100 µL aliquots were transferred to fresh LB medium and incubated at different pH (4, 6, 8, and 10), temperature (4, 20, 37, 45, and 60 °C), NaCl or LiCl (0.0, 0.5, 1.0, and 2.0 M). Growth under each condition was followed by changes in absorbance at 620 nm, and the specific growth rates were tabulated at the exponential phase of growth. In addition, the effect of Na or Li additions on the growth rate of *E*. *coli*, *S*. *aureus*, *S*. *epidermidis,* and *S*. *saprophyticus* was also evaluated.

*Genomic analysis.* Draft genome of *Staphylococcus sciuri* strain LCHXa was retrieved from NCBI, accession number NADO00000000 [14]. An improvement of the draft genome was made by using the genome of *S*. *sciuri* FDAARGOS-285 and *S*. *sciuri* SNUDS-18 as a guide for ordering the contigs into a chromosome. RAST subsystem was used to classify genes into metabolic categories. The phylogenetic analysis of the 16S rRNA gene tree was done by ClustalW in MEGA 7 [19]. The evolutionary history was inferred by using the Neighbor Joining method, and sequences for the tree were retrieved from the NCBI database. Sequence alignments of *gbs*A and *gbs*B genes were done by standalone blast against *Bacillus subtilis* subsp. *subtilis* str. 168 corresponding genes, accession number NC_000964.3.

*Purification of glycine betaine (GB)*. GB was extracted according to previously reported protocols [20,21]. Briefly, LCHXa cells were grown in LB medium in the presence or absence of 1 M NaCl or 1 M LiCl. The cell pellets were recovered by centrifugation (IEC/Centra CL2, 1957× *g*, 10 min, room temperature), suspended in water (HPLC grade), and freeze-dried. Twenty-five milligrams of dried biomass were exhaustively mixed with 570 µL of methanol/chloroform/water (10/4/4) for 5 min; next, 170 µL of chloroform and 170 µL water were added and mixed. Phase separation was obtained by centrifugation (IEC/Centra CL2; 5000 rpm, 5 min, room temperature). The upper aqueous phase was recovered, an aliquot was mixed with four volumes of acetonitrile (Merck, HPLC grade), and GB identification and content were conducted by HPLC. Fifty microliters of this solution were injected into a Shimatzu Hitachi LC-20A Prominence HPLC system (Cromtek, Huechuraba, Santiago, Chile) containing a C18 reverse phase analytical column (Merck, Nucleosil 100-10SA, 10 µm, 250 × 4.6 mm) as the stationary phase. For elution, the mobile phase used was KH_2_PO_4_/water (50:50, *v/v*) at a flow rate of 1.2 mL/min for 20 min. The HPLC fractions were recovered, dried, and dissolved in 100% methanol. Standards of GB, glycerol, ectoine and proline were prepared in HPLC quality water and run under the same conditions.

*GB quantification*. Standard GB (Sigma) was chromatographed in triplicate, in the (Cromtek, Huechuraba, Santiago, Chile) Shimatzu Hitachi LC-20A Prominence HPLC system, under the conditions previously described, using the following GB concentrations: 0.0, 37.5, 75.0, 150.0, and 300.0 µg GB. The area under the GB peaks was obtained with the program Labsolutions LC/GC version 5.57, included in the HPLC system. Next, a calibration curve (area versus µg GB) was prepared, and the corresponding equation (Y = 72.795 X; R^2^: 0.9549) was used to determine the GB content in each experimental sample.

*Scanning electron microscopy*. Strain LCHXa was grown in LB medium with and without 1 M LiCl, and cells were harvested at the exponential phase of growth. Freeze-dried cells were placed onto carbon strips, gold coated, and observed with a JSM-T300 JEOL microscope ((JEOL Ltd., Tokyo, Japan).

*Statistical analysis*. Statistical data analysis was done by ANOVA with Bonferroni correction (confidence level: 95%, *p* ≤ 0.05).

## 3. Results

### 3.1. Sites of Collection

**Figure 1:**
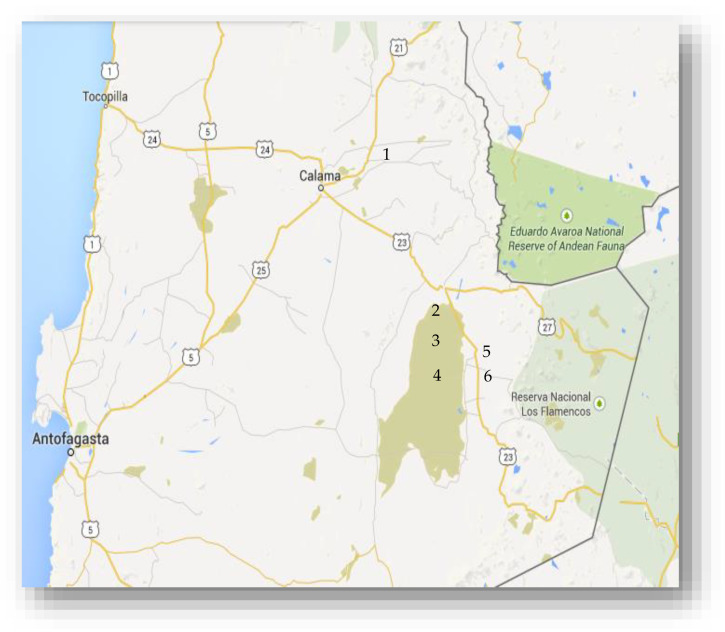
Sampling sites at Salar de Atacama and surrounding areas. Lakes Cejar (2), Tebenquiche (3), and Chaxa (4) are located within Salar de Atacama (greenish area). Chiuchiu Pond (1), and ravines Talabre (5) and Camar (6) are samplings sites at various distances from the Salar de Atacama basin. This map was obtained from Biblioteca del Congreso Nacional de Chile (https://www.bcn.cl/).

The 2014 sampling campaign at Salar de Atacama and surrounding areas included six sites located at the western slope of the Andes Range, above 2200 m of altitude (Figure 1). This campaign was centered on the search, isolation, and characterization of lithium-tolerant microorganisms. The selection of microorganisms was based on the microbial ability to grow in LB medium supplemented with 1 M LiCl. All sampling sites contained different concentrations of soluble lithium. At Salar de Atacama: Laguna Cejar (106 mg/L), Laguna Tebenquiche (429 mg/L), and Laguna Chaxa (1393 mg/L) and, at various distances from the salar: Chiuchiu Pond (8.2 mg/L), Talabre ravine (0.07 mg/L), and Camar ravine (0.81 mg/L) (Figure 1). The presence of free-living Li-tolerant microorganisms was observed in all the samples. Strain LCHXa, with growth in 1 M LiCl LB medium, was selected for further work, considering that it was isolated from water samples taken at Laguna Chaxa, which has a 10% salinity and the highest lithium concentration (33 mM LiCl).

### 3.2. Morphology of Strain

**Figure 2:**
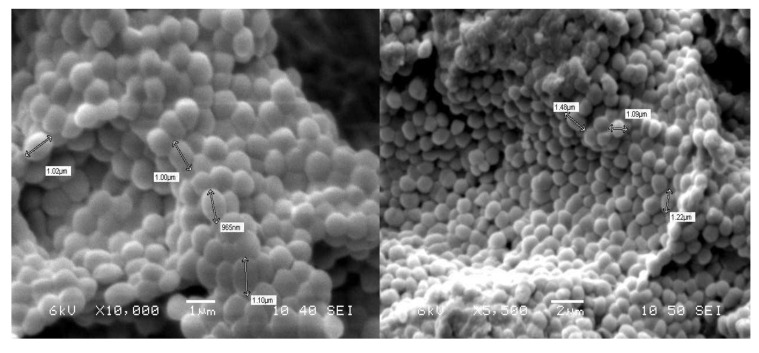
SEM micrographs of Staphylococcus sciuri strain LCHXa grown in Luria-Bertani (LB) medium (left) and LB medium supplemented with 1 M LiCl (right).

LCHXa. Isolate LCHXa is a non-motile, Gram-positive, cluster-forming coccus. It forms bright, yellowish-white, round colonies in LB medium, changing to colonies with irregularly undulated borders in the presence of 1 M LiCl. SEM measurement rendered mean diameters of 1.02 ± 0.06 μm for cells grown in LB medium and 1.24 ± 0.3 for cells grown in Li-LB medium (Figure 2).

### 3.3. Phylogeny of Strain LCHXa

**Figure 3:**
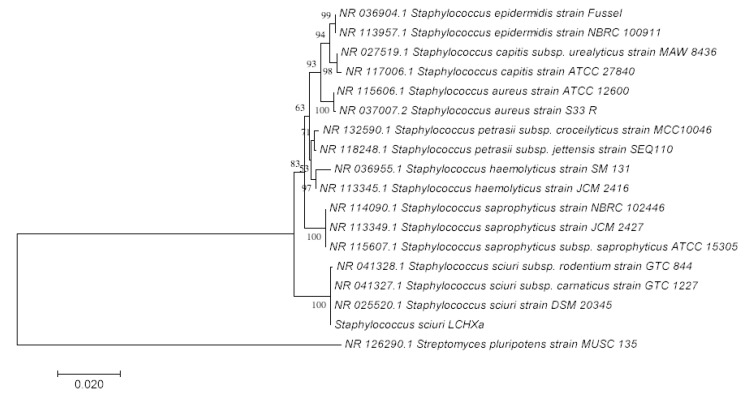
Phylogenetic analysis of 16S rRNA gene sequences by the Neighbor Joining method. Evolutionary analyses were done with MEGA7. The horizontal bar at the base of the figure represents 0.02 substitutions per nucleotide site. The percentage of trees for associated taxa is shown at the branches, with a bootstrap of 1000.

The alignments performed on standalone BLAST showed an identity above 99% between S. sciuri strain LCHXa and S. sciuri strain DSM20345, S. sciuri subsp. carnaticus strain GTC 1227, and *S*. *sciuri* subsp. *rodentium* strain GTC 844. The phylogenetic analysis by the Neighbor Joining method based on 16S rRNA is shown in Figure 3.

### 3.4. Metabolic Capabilities of Staphylococcus sciuri Strain LCHXa

Biochemical tests were performed in order to provide a metabolic insight on strain LCHXa and to support its phylogeny. Strain LCHXa was coagulase negative, but it rendered positive to catalase, oxidase, casein hydrolysis, and sugar fermentation tests (Table 1). The catalase test result was expected for members of the Staphylococcus genus but excluded LCHXa from the *Streptococcus* genus. In addition, the coagulase-negative result was indicative that LCHXa was not *Staphylococcus aureus*.

### 3.5. Antibiotic Susceptibility of Staphylococcus sciuri Strain LCHXa

The phylogenetic and metabolic analyses carried out previously showed that strain LCHXa belongs to the genus *Staphylococcus*. Since resistance/susceptibility to novobiocin is particularly important for taxonomic purposes [22], growth of strain LCHXa was challenged with 26 antibiotics and evaluated by the disc diffusion test in cells cultured in Mueller-Hinton agar plates. Based on the inhibitory zone diameters, strain LCHXa was resistant to 11 antibiotics, including novobiocin (Table 2).

The effect of salinity observed on the antibiotic resistance/susceptibility of LCHXa cells indicated that (i) cells grown in the presence of 1 M NaCl lost their resistance to cefuroxime, with a change in the inhibition zone diameter from 16 to 24 mm; (ii) in the presence of 1 M NaCl, there was an increment of the inhibition zone diameter for five antibiotics (lincomycin, clindamycin, penicillin, ceftazidime, and novobiocin) but all were within the range accepted for resistance to these antibiotics [18]; (iii) with the exception of cefuroxime, the response of *Staphylococcus sciuri* strain LCHXa to all other antibiotics was similar to cells tested without 1 M NaCl; (iv) strain LCHXa grown in 1 M LiCl became susceptible to clindamycin, ampicillin, fosfomycin, and amoxicillin, maintained its resistance to novobiocin and cefuroxime, and its response to other antibiotics was the same as cells grown without lithium (Table 2).

### 3.6. Optimal Temperature and pH for Growth of Staphylococcus sciuri Strain LCHXa

Growth of LCHXa in LB medium was optimal at pH 6 to 10, showing a nearly 50% decrease in its specific growth rate in the presence of 1 M NaCl. Addition of 1 M LiCl affected the LCHXa growth rate to a lesser degree at pH 6 to 8 (30%), but a serious decrease at pH 10 was observed (80%). Optimal growth of strain LCHXa in LB medium was observed at 20 to 37 °C. The specific growth rate of LCHXa was maximal at 37 °C in the presence of 1 M NaCl or 1 M LiCl but was 20% lower than the growth in LB medium without the presence of any of these two salts.

### 3.7. Effect of Sodium or Lithium Chloride on Staphylococcus sciuri Strain LCHXa Growth

Laguna Chaxa has a salinity of 10% (*w/v*) and the highest lithium content (1393 mg/L), as recorded in our 2014 sampling expedition at Salar de Atacama. Thus, the effect of increasing molar concentrations of NaCl or LiCl on strain LCHXa growth was studied. The isolate was able to grow in LB medium supplemented with an ample range of salts concentrations, from 0.0 to 2.0 M NaCl or LiCl (Table 3).

Optimal growth was attained in the absence of any salt addition; the specific growth rate of isolate LCHXa decreased by 76% or 85% at 2 M NaCl or 2 M LiCl, respectively. Then, *Staphylococcus sciuri* strain LCHXa is a novel mesophilic bacterium with an ample pH range for growth and is the first free-living, halotolerant, and lithium-tolerant *Staphylococcaceae* isolated from Salar de Atacama, at the Atacama Desert.

The tolerance of strain LCHXa to 1 M sodium or lithium chloride was compared with several *Staphylococci* species and *E. coli* (Table 4). All Staphylococcus strains showed tolerance to high NaCl or LiCl salinity. The most susceptible species to lithium were *S. saprophyticus* and *E. coli*. In contrast, *S. epidermis*, *S. aureus*, and strain LCHXa were more tolerant 1 M LiCl, showing a 69–78% decrease in their specific growth rate. *S. epidermidis* was the most tolerant species with only a 20% decrease in its specific growth rate in LB medium supplemented with 1 M LiCl.

### 3.8. An Insight on the Osmoregulatory Mechanisms of Staphylococcus sciuri Strain LCHXa

The draft genome sequence of *Staphylococcus sciuri* strain LCHXa has been recently reported to contain 58 protein-coding genes related to stress response and 17 protein-coding genes linked to osmoregulation [14]. We identified these 17 genes involved in response to osmotic stress, and 16 of them were associated to the capture, transport, or biosynthesis of glycine betaine (GB) (Table 5).

GB can be naturally found in plants, animals, and microorganisms, and it has been demonstrated that GB metabolism is directly related to osmotic stress tolerance in bacteria. Bacillus subtilis uses glycine betaine as an osmoprotector, and the *gbs*AB operon codes for glycine betaine dehydrogenase and alcohol dehydrogenase. The *gbs*AB operon allows the production of glycine betaine from choline in a two-step reaction: first, choline is oxidized to betaine aldehyde and, second, betaine aldehyde is dehydrated into glycine betaine [23,24].

Considering that GB biosynthesis in Bacillus subtilis was associated with the expression of dehydrogenase-coding genes *gbs*A and *gbs*B [23,24,25], the search for homologous genes in the LCHXa genome was conducted. Three open reading frames (ORF) with functions homologous to the gene cluster observed in *Bacillus subtilis* (i.e., *gbs*A, *gbs*B, and *gbs*R) were found in the LCHXa genome (Table 6). Therefore, we infer that the *gbs*AB operon is also present in *S*. *sciuri* strain LCHXa, and GB would be part of the tolerance response of strain LCHXa to osmotic stress.

The bioinformatic evidence obtained from the LCHXa genome prompted us to hypothesize that GB was key in the osmoregulation of S. sciuri strain LCHXa under molar concentrations of sodium or lithium. Then, the intracellular presence and content of GB in cells grown in the presence or absence of 1M NaCl, 1M LiCl, or 2 M LiCl were investigated.

**Figure 4:**
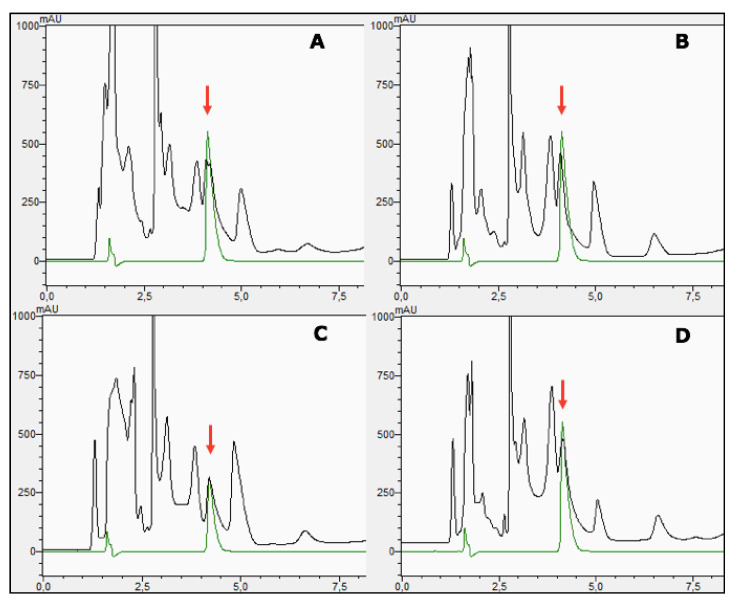
HPLC chromatograms of aqueous extracts from *S*. *sciuri* strain LCHXa. Biomasses of LCHXa cells were grown in LB medium without (**A**) or with the addition of 1M LiCl (**B**), 2M LiCl (**C**), and 1 M NaCl (**D**), and were harvested, freeze-dried, suspended in water, and compatible solutes were extracted from 25 mg of lyophilized biomass. Aliquots of 50 µL of extract were chromatographed and recorded at 198 nm. In order to visualize glycine betaine (GB) migration, independent standard GB (Sigma) chromatograms are included as green lines and arrows. Standard GB chromatograms were prepared with 37.5 ug GB at 4C and 75 µg GB at 4A, 4B, and 4D.

Based on the HPLC chromatographic profiles, all cell extracts showed the presence of GB with differences in content. The retention time (RT) for standard GB was 4.1 min (Figure 4). The average GB contents of strain LCHXa, expressed as mg g^−1^ dry biomass, were 34.3 ± 6.9 in LB without additions, 34.5 ± 10.5 in LB plus 1M LiCl, 26.6 ± 2.9 in LB plus 2 M LiCl, and 43.0 ± 7.8 in LB plus 1M NaCl. Then, GB content showed a relatively small difference between cells grown in the absence or presence of lithium; however, cells grown in 1M NaCl increased their GB content by nearly 25%.

The presence of glycerol was also identified in LCHXa cells by its HPLC migration. Standard glycerol migrated at RT 2.8 min coinciding with one of the stronger chromatographic signals with an average content of 269.5 ± 1.1 (LB without additions), 243.0 ± 2.6 (LB plus 1 M LiCl), 239.5 ± 1.4 (LB plus 2 M LiCl), and 251.5 ± 1.1 (LB plus 1 M NaCl) mg g^−1^ dry biomass. Comparatively, the glycerol content in LCHXa cells grown in LB medium is nearly eight-fold higher than the GB content. The minor differences in glycerol content under the different growth conditions suggest that glycerol may also contribute to osmoregulation, but its major role is probably in the cell metabolism.

Finally, the HPLC profiles of the cell extracts showed two additional signals. First, the signal peak at RT 3.8 min showed a similar area between cells grown in LB medium without or in the presence of lithium; however, there was a 50% area increase in cells grown in LB plus 1M NaCl, compared with the contents of cells grown with or without LiCl additions. Second, a prominent signal peak was observed at RT 4.9 min. The area of this signal peak showed a close similarity between cells grown in LB medium and 1 M LiCl medium; however, cells grown in 2 M LiCl increased in nearly 50% the area of this signal, in comparison with cells grown in LB medium; but cells grown in 1 M NaCl showed a 50% area decrease at RT 4.9 in comparison with cells grown in LB without additions. Then, the area of the peak at 4.9 min in cells grown in 2 M LiCl was three-fold higher than the area from cells grown in 1 M NaCl. As a preliminary identification of the signal peaks with RT 3.8 min and 4.9 min, standards of L-proline and ectoine were chromatographed under the same experimental conditions. Their RTs were 3.4 min for L-proline and 3.01 min for ectoine, rejecting them as probable compatible solute candidates.

## 4. Discussion

*Staphylococcus sciuri* comprises more than 50 species and subspecies; taxonomically, it is considered the most primitive among *Staphylococci*; it is found primarily on rodents and primitive mammal; it has been isolated from soil, sand, and natural waters, and its clinical relevance for humans has been previously stressed [22,26,27].

Lithium-tolerant microorganisms. Although there is limited information on isolation and characterization of microorganisms tolerant to high concentrations of lithium, there is an increasing trend of interest on them [8,9,10,11,12,13,14]. For example, Kamekura and Onishi [8] recovered spontaneous lithium-resistant mutants of *Micrococcus varians* ssp. *halophilus* ATCC 21971, which could grow at 1.5 M LiCl; Tsuruta [9] studied the ability of 70 strains of 63 species of microorganisms to adsorb lithium from a solution containing 75 µM Li, showing that Gram-positive bacteria were most effective. In addition, three *Staphylococcus aureus* strains and one *Staphylococcus warneri* strain were isolated from highly polluted water samples from River Kizilirmak, in Turkey [28], which were cataloged as lithium-resistant strains since they were able to grow in LB medium supplement with 0.12 M LiCl. Also, UVR-exposed *Rhodococcus* sp. A5wh rendered lithium-tolerant cells with 1.5 M LiCl as the limiting concentration for growth [11]. Members of the families *Halobacteriaceae*, *Rhodothermaceae,* and *Staphylococcaceae* have been found, and three lithium-tolerant *Bacillus* have been isolated from artificial solar evaporation ponds at Salar de Atacama [12,13].

*Staphylococcus sciuri* strain LCHXa as a lithium-tolerant microorganism. Salar de Atacama contains lithium-rich brines at 30–50 m deep, which have been under intensive industrial exploitation for several decades. After pumping the underground brines to artificial solar evaporation ponds, lithium salts are recovered, dried, and commercialized [6,29]. In our 2014 sampling campaign at and near Salar de Atacama, the search, isolation, and characterization of lithium-tolerant microorganisms were conducted at sites commercially unexploited. Microorganisms were selected by their ability to grow in LB medium supplemented with 1 M LiCl. Strain LCHXa was selected among several other isolates, since it was obtained from water samples at Laguna Chaxa, a lithium-rich natural water body within the Salar de Atacama, without commercial exploitation.

Strain LCHXa is a free-living, non-motile, lithium-tolerant, Gram-positive coccus belonging to the *Staphylococcus* genus, with a proximity to the *sciuri* species. Based on its genomic identity to *S*. *sciuri* FDAARGOS 258 and *S*. *sciuri* SNDS 18, strain LCHXa was named *Staphylococcus sciuri* strain LCHXa [14]. In this work, we have provided further metabolic information to support the assigned phylogeny of isolate LCHXa to the *Staphylococcus* genus.

The novel *Staphylococcus sciuri* strain LCHXa is a mesophilic bacterium with optimal growth at 37 °C in LB medium. The addition of 1 M NaCl or 1 M LiCl negatively affected its specific growth rate, maintaining the optimal growth temperature. Also, optimal growth was observed at pH 6–10 for cells grown in LB medium. Strain LCHXa cells grew in the presence of 1 M NaCl at the same pH range with a 20% drop in their specific growth rate. A pH of 6–8 was optimal for growth in 1 M LiCl, and a drastic decline in specific growth rate was observed at pH 10. Our study showed that *Staphylococcus sciuri* strain LCHXa could grow in the presence of LiCl or NaCl, at an ample range of concentration from 0.0 to 2 M. Halotolerance was evident since the microorganism grew better in LB medium without salt additions. Under the highest salt concentration tested, *Staphylococcus sciuri* strain LCHXa diminished its specific growth rate by 75% (2 M NaCl) and 85% (2 M LiCl).

Growth of *Staphylococcus aureus* at high osmolarity is improved in the presence of choline, glycine betaine, proline, or taurine [30]. Halotolerance in *Staphylococci* has been recently reviewed with emphasis on cell wall modifications, cytoplasmic modulation, and antibiotic susceptibility under osmotic stress [31]. Cell volume plasticity of *S*. *sciuri* strain LCHXa was observed under SEM microscopy as a 20% increase in cell diameter after growth in LB supplemented with 1 M LiCl. This morphological response to high osmotic pressure may be correlated to transport or biosynthesis of compatible solutes, increasing the intracellular solute concentration [32].

A comparison of lithium tolerance between *Staphylococcus sciuri* strain LCHXa and other bacteria (*S*. *saprophyticus*, *S*. *epidermidis*, *S*. *aureus,* and *E*. *coli*) showed that all the *Staphylococci* species tested were tolerant to 1 M LiCl, with a reduction of 70–80% on their specific growth rate. *Staphylococcus epidermidis* was most tolerant, showing a 20% decrease in growth rate at 1 M LiCl and showed an improved growth at 1 M NaCl. *E*. *coli* cells showed the best growth in LB medium, and it could sustain growth under 1 M NaCl (70% decrease in growth rate), but it was unable to grow in 1 M LiCl (98% decline in growth rate).

Metabolic characterization of *Staphylococcus sciuri* strain LCHXa. Isolate LCHXa was a coagulase-negative, indole-negative, catalase-positive microorganism, able to ferment several carbohydrates (e.g., glucose, lactose, xylose). The microorganism was resistant to 11 antibiotics, including novobiocin, oxacillin, and penicillin. However, cells grown under osmotic stress showed changes in resistance/susceptibility to some antibiotics. Loss of the resistance to cefuroxime was the only change observed in LCHXa cells grown at 1 M NaCl. However, in the presence of LiCl, LCHXa cells became susceptible to five antibiotics (Clindamycin, Ampicillin, Fosfomycin, Oxacillin, Amoxicillin) and resistant to Cefuroxime. Resistance to Novobiocin was maintained under both osmotic stresses. A recent review of the adaptability strategies to osmotic stress and susceptibility to antibiotics of *Staphylococci* species showed that *S*. *aureus* and *S*. *epidermidis* are susceptible to Novobiocin but *S*. *saprophyticus* and *S*. *sciuri* are resistant [31]. Our search on the genome of *Staphylococcus sciuri* strain LCHXa published recently by Vilo et al. [14] showed the presence of the gene mecA (data not shown), in agreement with studies on this microbial group [27,33,34,35].

Osmoregulation in *Staphylococcus sciuri* strain LCHXa. The genome of strain LCHXa contains 58 protein-coding genes related to stress response [14]. We have identified 16 of them associated with the capture, transport, or biosynthesis of glycine betaine. One of these genes was coded for a protein associated with the uptake of glycerol, and the other 16 genes were involved in the capture, transport, or biosynthesis of GB. Also, three ORF were identified in the LCHXa genome with homologous functions to genes *gbs*A, *gbs*B, and *gbs*R from *B*. *subtilis*, which code for betaine aldehyde dehydrogenase, alcohol dehydrogenase, and the betaine operon transcription regulator. Interestingly, we observed that *gbs*ABR ORF observed in strain LCHXa were forming a cluster in the same order of *B*. *subtilis* operon, which is indicative of its function in osmoregulation [23,24,25].

The presence of GB was confirmed by HPLC in all cell extracts from the strain LCHXa; the GB content ranged from 34 to 43 mg g^−1^ dry biomass, depending upon de the presence/absence of NaCl or LiCl in the growth medium. In the presence of 1 M or 2 M LiCl, the GB content was like that of cells without Li addition. In contrast, growth in 1 M NaCl improved GB content by 25%. Previous reports have shown that GB and proline are compatible solutes used by *S*. *aureus* to sustain growth at molar NaCl concentrations [36,37,38,39]. Osmoregulation studies on *Staphylococcus aureus* mutants lacking a low-affinity transport system and grown under extreme osmotic stress caused by 4 M NaCl or 3.5 M LiCl provided several conclusions: (a) there were no changes in the cell adenylate energy charge, and growth was not impaired by ATP as a limiting factor; (b) high lithium or sodium concentrations were toxic to both strains with a rapid decrease in cell numbers, more accentuated under Li, and not as a consequence of an osmotic effect; (c) the effect of high lithium concentration, as a chaotropic molecule, would cause protein precipitation upon entering the cells; and (d) high-affinity transports systems for proline and glycine betaine play a role in osmoregulation in *S*. *aureus* strains under extreme osmotic stress [38]. Comparatively, 2 M LiCl or 2 M NaCl were the highest concentrations used in our experiments with *Staphylococcus sciuri* strain LCHXa, and the strain could still grow.

Finally, the absence of chromatographic signals detecting the presence of proline or ectoine in all the LCHXa extracts would exclude these osmolytes from the probable mechanism of osmoregulation in *Staphylococcus sciuri* strain LCHXa. Considering that the content of glycine betaine did not change substantially in the presence or absence of 1 M or 2 M LiCl but increased by 25% during growth at 1 M NaCl, we suggest that GB would be a constitutive osmoprotectant in *Staphylococcus sciuri* strain LCHXa, probably complementing the role of glycerol in the osmoregulatory response of strain LCHXa. Future work will be focused on the identification of other compatible solutes involved in the response of *Staphylococcus sciuri* strain LCHXa to osmotic stress.

## 5. Conclusions

*Staphylococcus sciuri* strain LCHXa is the first free-living member of the *Staphylococcus* genus isolated from the nonindustrial, natural Laguna Chaxa at Salar de Atacama basin in northern Chile. Strain LCHXa is tolerant to molar concentrations of LiCl, and its osmoregulatory mechanism involved the synthesis of glycine betaine and glycerol as constitutive osmoprotectants, as well as other compatible solutes still unidentified.

## Figures and Tables

**Table 1:** Biochemical tests done on strain LCHXa.

Biochemical Test	Strain LCHXa
Simmons citrate	-
Mobility	-
Indole	-
Ornitina	-
TSI	A/A
LIA	K/K
Coagulase	-
Catalase	+
Oxidase	-
Starch hydrolysis	-
Casein hydrolysis	+
Tween 60 hydrolysis	-
Tween 80 hydrolysis	-
DNA hydrolysis	+
Glucose fermentation	+
Lactose fermentation	+
Mannitol fermentation	+
Xylose fermentation	+
Cellobiose fermentation	+
N-acetylglucosamine fermentation	-
Hemolysis	β
+: positive reaction; -: negative reaction;.β: beta hemolysis; A: acid; K: base.	

**Table 2:** Susceptibility of *Staphylococcus sciuri* strain LCHXa to 26 antibiotics. Cells were grown in Mueller-Hinton agar plates (MH), in the presence of 1 M NaCl (MH-Na) or 1 M LiCl (MH-Li) and incubated at 35 °C for 24 h in the presence of disc-containing antibiotics. Diameter of the inhibition zones and their interpretation were done according to the manufacturer’s instruction [18]. nt: not tested. R: resistant; S: susceptible. Numbers in parentheses represent the radius of the inhibition halos in millimeters.

Antibiotic	Potency (μg)	Susceptibility (Inhibition Zone in mm)		
		MH	MH-Na	MH-Li
Ampicillin/Sulbactam	10/10	R (0)	R (0)	R (27)
Lincomycin	2	R (13)	R (16)	R (16)
Clindamycin	2	R (0)	R (11)	S (23)
Penicillin	10	R (0)	R (10)	R (21)
Ampicillin	10	R (0)	R (0)	S (29)
Ceftazidime	30	R (0)	R (10)	R (0)
Fosfomycin	200	R (0)	R (0)	S (40)
Oxacillin	1	R (0)	R (0)	S (14)
Amoxicillin	20/10	R (14)	R (14)	S (31)
Novobiocin	5	R (0)	R (16)	R (10)
Cefuroxime	30	R (16)	S (24)	R (11)
Gentamicin	10	S (30)	S (25)	S (25)
Tetracycline	30	S (26)	S (30)	S (22)
Vancomycin	30	S (20)	S (24)	S (21)
Cotrimoxazole	25	S (28)	S (32)	nt
Erythromycin	15	S (22)	S (30)	nt
Ciprofloxacin	5	S (34)	S (30)	S (31)
Chloramphenicol	30	S (24)	S (28)	nt
Neomycin	30	S (21)	S (20)	nt
Nitrofurantoin	300	S (25)	S (29)	S (22)
Cefadroxil	30	S (18)	S (15)	S (29)
Streptomycin	300	S (28)	S (22)	S (26)
Levofloxacin	5	S (31)	S (30)	S 30)
Teicoplanin	30	S (17)	S (20)	nt
Linezolid	30	S (30)	S (32)	nt
Amikacin	30	S (26)	S (22)	S (23)

**Table 3:** Effect of NaCl and LiCl on the specific growth rate of *Staphylococcus sciuri* strain LCHXa. Cells were grown in LB medium supplemented with an ample range of salt concentrations. Results are shown as mean and standard deviation (n: 3).

Concentration (M)	Specific Growth Rate (h^−1^)	
	NaCl	LiCl
0.0	0.41 ± 0.01	0.41 ± 0.01
0.2	0.35 ± 0.01	0.39 ± 0.01
0.4	0.35 ± 0.01	0.34 ± 0.03
0.8	0.32 ± 0.04	0.29 ± 0.01
1.0	0.33 ± 0.02	0.31 ± 0.02
1.5	0.19 ± 0.04	0.18 ± 0.02
2.0	0.10 ± 0.01	0.06 ± 0.01

**Table 4:** Specific growth rate of strain LCHXa, species of the *Staphylococcus* genus and *Escherichia coli*. Cells were grown in LB medium or LB medium supplemented with 1 M NaCl (Na-LB) or 1 M LiCl (Li-LB). Results are shown as mean ± standard deviation (n: 3). Numbers in parentheses show the percentage of the growth rate compared with the corresponding condition without salt addition.

Microorganism	Specific Growth Rate (h^−1^)		
	LB	Na-LB	Li-LB
*S*. *sciuri* strain LCHXa	0.58 ± 0.01	0.28 ± 0.01 (51)	0.13 ± 0.01 (78)
*S*. *saprophyticus*	0.26 ± 0.01	0.13 ± 0.02 (50)	0.08 ± 0.01 (69)
*S*. *aureus*	0.76 ± 0.03	0.31 ± 0.01 (59)	0.18 ± 0.01 (76)
*S*. *epidermidis*	0.24 ± 0.01	0.32 ± 0.07 (133)	0.19 ± 0.01 (21)
*E*. *coli*	1.42 ± 0.02	0.42 ± 0.23 (70)	0.03 ± 0.00 (98)

**Table 5:** Genes and gene copies involved in the response of *Staphylococcus sciuri* strain LCHXa to osmotic stress.

Function	Subsystem	Gene Copy
Glycerol uptake facilitator protein	Osmoregulation	1
Glycine betaine ABC transport system, glycine betaine-binding protein OpuAC	Choline and betaine Uptake and betaine biosynthesis	2
Osmotically activated L-carnitine/choline ABC transporter, permease protein OpuAC	Choline and betaine Uptake and betaine biosynthesis	1
Osmotically activated L-carnitine/choline ABC transporter, permease protein OpuCB	Choline and betaine Uptake and betaine biosynthesis	1
Glycine betaine ABC transport system, ATP binding protein OpuAA (EC 3.6.3.32)	Choline and betaine Uptake and betaine biosynthesis	2
Betaine aldehyde dehydrogenase (EC 1.2.1.8)	Choline and betaine Uptake and betaine biosynthesis	1
Glycine betaine transporter OpuD	Choline and betaine Uptake and betaine biosynthesis	3
Osmotically activated L-carnitine/choline ABC transporter, substrate-binding protein OpuCD	Choline and betaine Uptake and betaine biosynthesis	1
L-proline glycine betaine binding ABC transporter protein ProX (TC 3.A.1.12.1)	Choline and betaine Uptake and betaine biosynthesis	1
Osmotically activated L-carnitine/choline ABC transporter, substrate-binding protein OpuCC	Choline and betaine Uptake and betaine biosynthesis	2
Choline dehydrogenase (EC 1.1.99.1)	Choline and betaine Uptake and betaine biosynthesis	1

**Table 6:** Alignments results of *Staphylococcus sciuri* strain LCHXa genes with homologous functions to genes *gbs*A y *gbs*B from *B*. *subtilis*.

B. Subtilis Gene	Metabolic Function	% Identity	Size (bp)
gbsA (Locus tag: BSU_31060)	Betaine aldehyde dehydrogenase	66%	1490
gbsB (Locus tag: BSU_31050)	Choline dehydrogenase	30%	1682
gbsR (Locus tag: BSU_31070)	Betaine operon transcriptional regulator	57%	554
